# Examining factors affecting female suicide and solutions to cope with these factors: a review study

**Published:** 2019-08

**Authors:** Ali Saljoghipebdeni, Heshmatolah Mannsouri Bidekani, Sedigheh Heydari Soureshjani, Lela Ghasemi Dastgerdi, Sosan Asghari Andehkordi

**Affiliations:** ^*a*^Local Health Center of Koohrang, Koohrang, Iran.; ^*b*^Hajar Hospital of Nursing, Shahrekord University of Medical Sciences, Shahrekord, Iran.; ^*c*^Department of Health, Shahrekord University of Medical Sciences, Shahrekord, Iran.

## Abstract

**Background:**

Suicide is of one the leading causes of death in the world. Increasing rate of suicide among women has been reported many times. Women constitute one of the most important pillars of the society and families and the health of society and family is dependent on meeting their health, cultural, and economic needs. In this regard, the researcher has studied factors affecting female suicide and solutions to cope with these factors.

**Methods:**

In this study, articles published in English and Persian languages in scientific sites, psychological journals and conferences of medical sciences, psychology and psychiatry, SID, PubMed and Scopus, and humanities were searched by keywords such as women safety, emotional, physical and sexual needs of women, suicide, and factors affecting suicide and also in-person reference to forensic medicine in Chahar Mahal and Bakhtirai province.

**Results:**

Investigations show that family problems, divorce, depression, hopelessness, disease, illegal relationships or rape, alcohol and drugs, personal dependence, economic problems or unemployment, social pressures, love failure, emotional problems, and death of relatives are among the factors that cause female suicide. According to the information obtained from forensic medicine, most female suicides are occurred during menstruation that should be studied separately. Also, according to the findings, it seems that if females can form a family and have peace (from emotional and economic aspects), suicide rate decreases significantly.

**Conclusions:**

According to the findings, the most important suicide factors of Iranian women are family problems or lack of family formation and illegal relationships. For this purpose, it is necessary to increase awareness of people about marriage and provide facilities and encourage people and families to refer to family counseling centers if any problem occurs.

**Keywords:**

Females, Suicide, Family, Society

